# Developmental Expression of HSP60 and HSP10 in the *Coilia nasus* Testis during Upstream Spawning Migration

**DOI:** 10.3390/genes8070189

**Published:** 2017-07-21

**Authors:** Di-An Fang, Yan-Feng Zhou, Min-Ying Zhang, Dong-Po Xu, Kai Liu, Jin-Rong Duan

**Affiliations:** 1 Freshwater Fisheries Research Center, Chinese Academy of Fishery Sciences, Shanshui Road 9, Wuxi 214000, Jiangsu, China; zhouyf@ffrc.cn (Y.-F.Z.); zhangmy@ffrc.cn (M.-Y.Z.); xudp@ffrc.cn (D.-P.X.); liuk@ffrc.cn (K.L.); duanjr@ffrc.cn (J.-R.D.); 2 Scientific Observing and Experimental Station of Fishery Resources and Environment in the Lower Reaches of the Yangtze River, Ministry of Agriculture, Xuejiali 69, Wuxi 214000, Jiangsu, China

**Keywords:** *Coilia nasus*, spawning Migration, heat shock protein 60, heat shock protein 10

## Abstract

Heat shock protein 60 (HSP60) and heat shock protein 10 (HSP10) are important chaperones, which have been proven to have essential roles in mediating the correct folding of nuclear encoded proteins imported to mitochondria. Mitochondria are known as the power house of the cell, with which it produces energy and respires aerobically. In this regard, the obtained HSP60 and HSP10 have typical characteristics of the HSP60/10 family signature. Their mRNA transcripts detected were highest during the developmental phase (in April), while the lowest levels were found in the resting phase (after spawning in late July). Additionally, the strongest immunolabeling positive signals were found in the primary spermatocyte, with lower positive staining in secondary sperm cells, and a weak or absent level in the mature sperm. At the electron microscopic level, immunogold particles were localized in the mitochondrial matrix. Data indicated that HSP10 and HSP60 were inducible and functional in the *Coilia nasus* testis development and migration process, suggesting their essential roles in this process. The results also indicated that HSP60 may be one indicator of properly working mitochondrial import and refolding in the fish testis. This study also provides an expanded perspective on the role of heat shock protein families in spawning migration biology.

## 1. Introduction

Anadromous fish spawning migration is a highly complex temporal event and a process which consumes energy [1,2]. *Coilia nasus* is an important kind of anadromous fish species, which is widely distributed in the sea as well as in fresh water such as the Yangtze River [3,4]. *C. nasus* spawning migration was classically considered a one-time seasonal reproductive activity [5,6]. Mature fish usually migrate upstream and spawn in the lower and middle reach of the Yangtze River in China, and then eggs float down and hatch near the river mouth [6]. During the fish spawning migration process, long distance swimming induced fish to consume more energy and encounter a lot of stress, such as water temperature and water flow, which results in up- or downregulation of numerous adaptive genes, including heat shock proteins (HSPs), to adapt to the migration behavior [7,8,9].

Most HSPs show a significant response to different forms of environmental stresses [10,11]. Among different forms of HSPs, HSP60 is a mitochondrial matrix protein, which is mainly located in the mitochondria of eukaryotic cells [12]. It is a molecular chaperone which influence the folding of other HSP [13]. HSP60/HSP10 are destined for the mitochondrial matrix, which are mainly encoded in the nucleus. In the matrix, HSP60/HSP10 are usually maintained in an unfolded state [12]. HSP60/HSP10 folding and release from complex aggregates requires hydrolysis of ATP [13]. Furthermore, it was suggested that HSP60/HSP10 are also regulated developmentally and expressed highly in gonads, and they were first described in rats [14]. In the sea bream, water temperature stress especially upregulated HSP10, HSP60 and HSP70, which could improve the oxidative capacity [15]. Further study results indicated that the ATP-hydrolytic activity of HSP60 is regulated by its co-chaperonin HSP10. Moreover, studies have suggested that HSP60 plays a key role in preventing apoptosis in the cytoplasm by forming a complex with HSP10, and regulates the activity of these proteins [12]. Also HSP10 aids HSP60 in protein folding by acting as a dome-like cover on the ATP active form of HSP60 [14]. The complex matrix HSP60/HSP10 is formed to fulfill mitochondrial protein folding during energy metabolism [15]. In recent years, more and more studies have focused on the matrix HSP60/HSP10 in mitochondria, which might be indispensable in a variety of developmental processes [15].

Mitochondria are cellular organelles that play important roles in eukaryotic cell physiology, ranging from ATP production and biosynthesis to cellular homeostasis, including spermatogenesis [16,17]. The mitochondria number and activity can both be modulated through the transcriptional and translational regulation of nuclear and mitochondrial proteins, which reflect the cell energy requirement [18]. Additionally, they play important roles in the regulation of mitochondrial protein transportation into the mitochondrial matrix from the cytoplasm [13].

Literature on the regulation of mitochondrial activity and biogenesis is poorer in fish species than in higher vertebrates, although it appears that fish mitochondria are especially versatile [14,19,20]. Fish mitochondrial activity is highly modulated by thermal or nutritional stresses [19,21]. Mitochondrial function is highly regulated by diet in gilthead sea bream *Sparus aurata*, but it remains unclear how these processes initiate retrograde signals for transcriptional regulation of mitochondrial biogenesis [15]. Previously, we have shown that during *C. nasus* spawning migration, the fish stomach is usually empty, while the process is a humungous energy consuming process. Served as a matrix of mitochondria, it is not withstanding that HSP60/HSP10 complex participates in the migration behavior and spermatogenesis. In this regard, this study aims to determine whether mitochondrial response could be used as an informative tool, to provide new insights to define the threshold level of natural conditions in anadromous fish. The main objectives of the present study were: (1) to clone the *Cn-HSP60/HSP10* gene; (2) to investigate temporal mRNA expression of *Cn-HSP60/HSP10* in testes during spawning migration; and (3) to locate Cn-HSP60/HSP10 protein matrix during different developmental phases of sperm in testes. Insight into the *Cn-HSP60/HSP10* gene and its expression during upstream migration process is important for understanding the molecular mechanism of anadromous fish gonad development and reproductive biology. Combined with data from other literature on fish developmental biology, results of this present study may facilitate further investigations on the spawning migration mechanism.

## 2. Results

### 2.1. Characteristics of HSP60 and HSP10 cDNA

**Figure 1:**
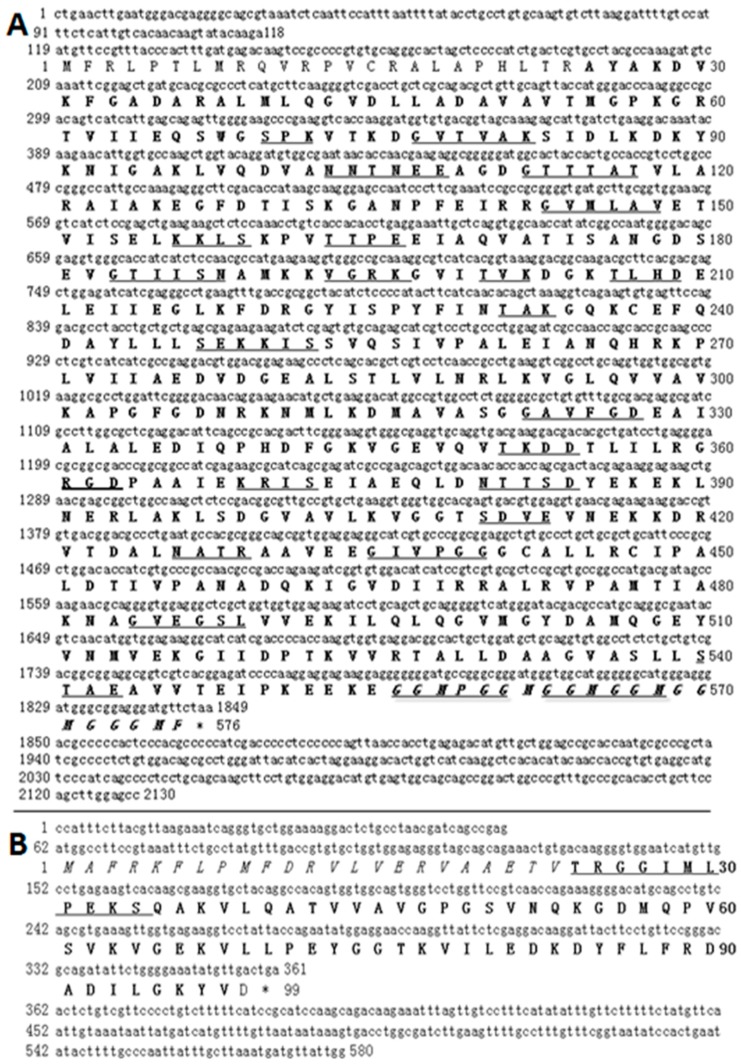
Nucleotide and deduced amino acid sequences of Cn-HSP60 and Cn-HSP10.The deduced amino acid sequence is shown upon the nucleotide sequence. The termination codon is marked by an asterisk. (**A**) is shown the Cn-HSP60: RGD Cell attachment sequence is shown in bold line. The conserved domain (26-571 aa, 60 kDa chaperonin (groEL) ) is in bold (Cpn60); Amidation site (193–196aa); ASN Glycosylation site (103–106 aa, 380–383aa, 426–429 aa); CAMP phosphorylation site (156–159 aa, 249–252 aa, 369–372 aa); CK2 phosphorylation site (105–108 aa, 163–166 aa, 200–203 aa, 206–209 aa, 351–354 aa, 381–384 aa, 410–413 aa, 540–543aa), Myristyl (77–82 aa, 112–117 aa, 143–148 aa, 183–188 aa, 322–327 aa, 435–440 aa, 484–489 aa, 556–561 aa, 563–568 aa, 569–574 aa); PKC phosphorylation site (70–72 aa, 200–202 aa, 231–233 aa, 247–249 aa) are underlined. The C-terminal Gly-Gly-Met (GGM) repeat is in italic. (**B**) is shown the Cn-HSP10: The putative mitochondrial targeting sequence is in italic. The conserved domain (GroES domain) is shaded in bold; the predicted mobile loop is underlined.

Using rapid amplification of cDNA ends (RACE) PCR method and comparing with genome database, the complete *Cn-HSP60* cDNA was 2130 bp in length, including an open reading frame (ORF) of 1728 bp, a 118 bp 5’-untranslated region (UTR), and a 281 bp 3’- UTR. The predicted ORF encoded a protein of 576 amino acids with a calculated molecular weight (Mw) of 61.1 kDa and a theoretical predicted isoelectric point (pI) of 5.76. As expected, predicted protein of Cn-HSP60 had several HSP60 family motifs or domains, including a mitochondrial presequence, a characteristic HSP60 family signature, an ATP binding site, and a terminal typical GGM repeat motif (Figure 1A). The *Cn-HSP60* cDNA and predicted protein sequence had been submitted to GenBank and the accession number is KY364899.

A full-length cDNA ofHSP10(580 bp) in *C. nasus* was identified, which contained a predicted ORF of 297 bp, beginning with a methionine codon at position 62 and ending with a TGA termination codon at position 361. The 3′-untranslated region is 219 bp in length from 362 to 580 bp (Figure 1B). The complete sequence GenBank accession number is KY364900. The encoded 99 amino acid polypeptide had a Mw of 10.9kDa and an pI of 7.94. *HSP60* gene and one *HSP10* gene are present in the genome as a head-to-head gene pair (*C. nasus* genome data unpublished). The HSP60 protein was comprised of a GroEL domain while the HSP10 protein was comprised of a GroES domain (Figure 1A,B).

### 2.2. Homology and Phylogenetic Analyses of HSP60 and HSP10

**Figure 2:**
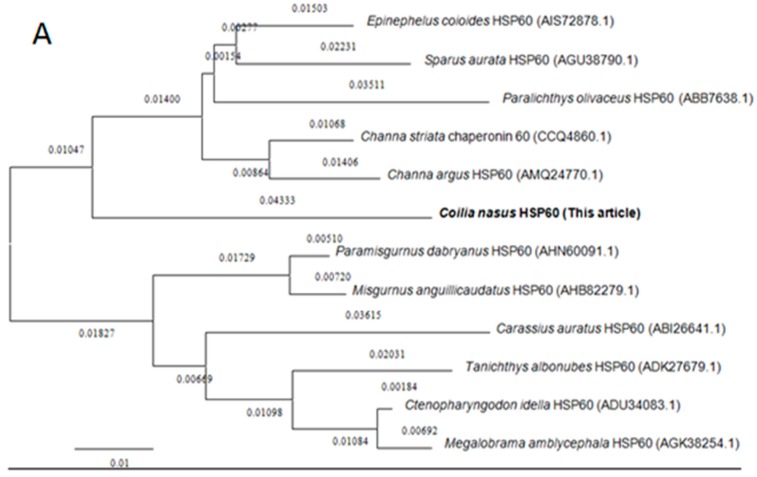

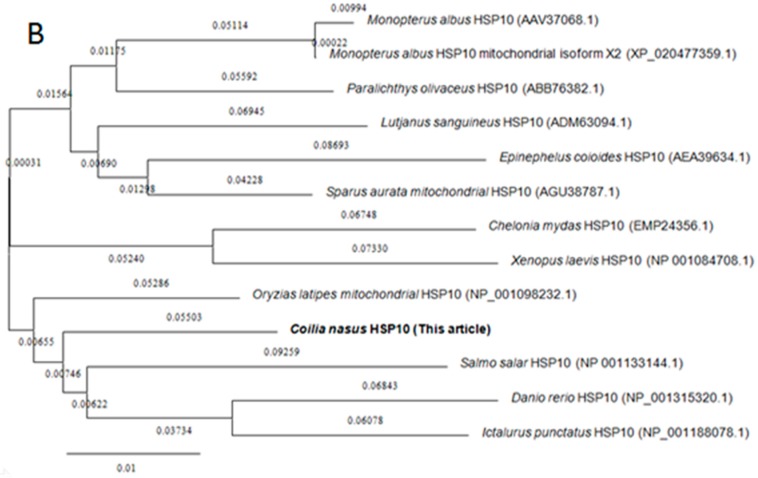
Phylogenetic trees of HSP60 and HSP10 family members. Phylogenetic tree constructed by the MEGA 4.0 program by the neighbor-joining distance method. (**A**) is for HSP60s and (**B**) for HSP10s. The statistical robustness of the tree was estimated by bootstrapping with 1000 replicates. Bootstrap values were indicated by genetic distance.

Amino acid sequences of HSP60 and HSP10 from different species were downloaded from the NCBI database for homology and phylogenetic analyses. BLAST analysis suggested that *C. nasus* HSP60 had higher sequence homology similarity with *Clupea harengus* (93%), *Channa argus* (89%), *Danio rerio* (89%), *Epinephelus coioides* (87%), *Paralichthys olivaceus* (87%), *Tanichthys albonubes* (86%), *Megalobrama amblycephala* (86%) and shared above 60% identity with other studied species’ HSP60s. HSP10 from *C. nasus* shared high homology with that from *Danio rerio* (96%), *Salmo salar* (96%), *Ictalurus punctatus* (95%), *Xenopus laevis* (89%), *Epinephelus coioides* (89%) and also shared above 60% identity with other studied species HSP10s. Phylogenetic trees were constructed by analyzing the amino acid sequences of *C. nasus* HSP60 and HSP10 with those from other species. Protein sequences of HSP60 and HSP10 were obtained from the NCBI data base, a neighbor-joining (NJ) phylogenetic tree indicated that the evolution of HSP60 and HSP10 was almost in accord with the evolution of species (Figure 2A,B).

### 2.3. HSP10and HSP60 mRNA Expression Patterns

**Figure 3:**
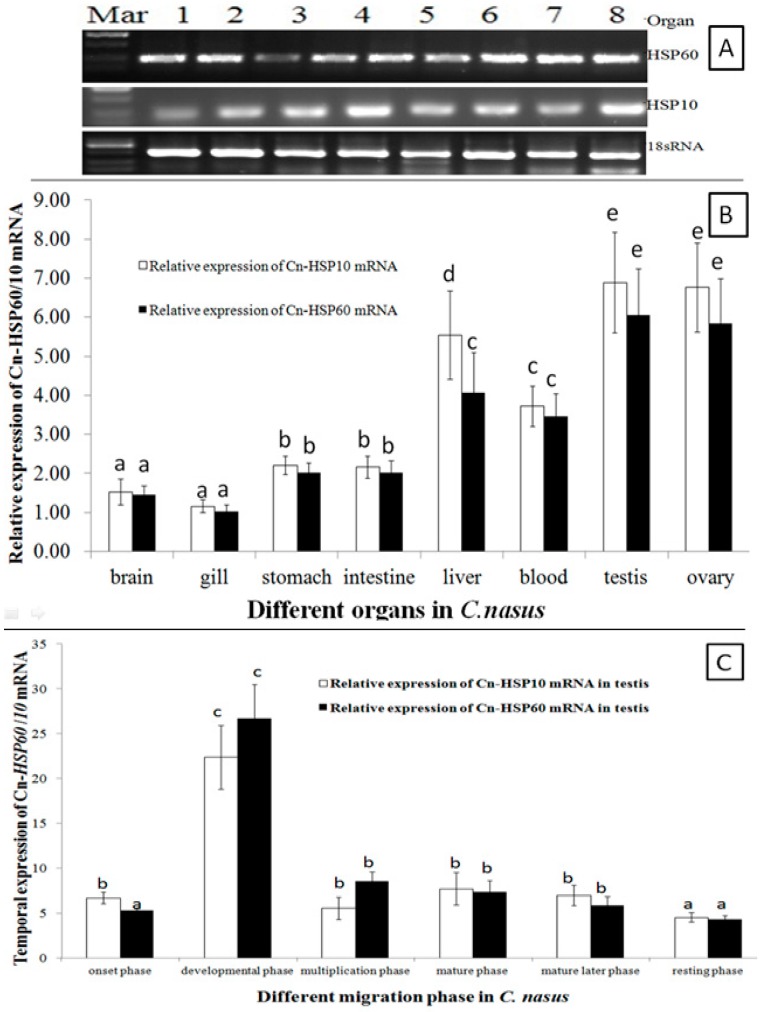
HSP60 and HSP10 mRNA Expression Patterns. (**A**,**B**) are for different organs by RT-PCR and RT-qPCR method, respectively; (**C**) is for different migration phase. Data were expressed as the mean fold difference (mean ± SE, pooled RNA, *n* = 6, one in each section, total *n* = 3 × 6 = 18). Expression values were normalized to those of 18sRNA. Values with the different superscript letters are significantly different (*p* < 0.05, a < b < c < d < e). In (**A**), Mar: molecular marker, 1: blood, 2: brain, 3: gill, 4: liver, 5: stomach, 6: intestine, 7: testis and 8: ovary. The different migration phases of the fish: onset phase (Chongming section in March), developmental phase (Nantong section in March to April), multiplication phase (Jingjiang section in April to May), mature phase (Zhenjiang section in May), mature later phase (Dangtu section in late May to early June), and resting phase (Anqing section in mid to late June).

*HSP60* and *HSP10* mRNA transcripts were universally expressed in all examined organs (Figure 3A). Expression level was significantly higher in gonads, liver and blood; with lower levels in brain, stomach and intestine, and the lowest levels were detected in the gill (Figure 3A,B). During the migration cycle in the gonad, the *HSP60* and *HSP10* mRNA transcripts temporal expression patterns were in a similar way during the migration cycle (Figure 3C). Transcripts of *HSP60* and *HSP10* mRNA were up-regulated to peak expression in the developmental phase, and significantly lower expression was maintained during the multiplication and mature period. Then, expression was downregulated, and the lowest expression level was found in the resting phase.

### 2.4. Western Blotting Results

**Figure 4:**
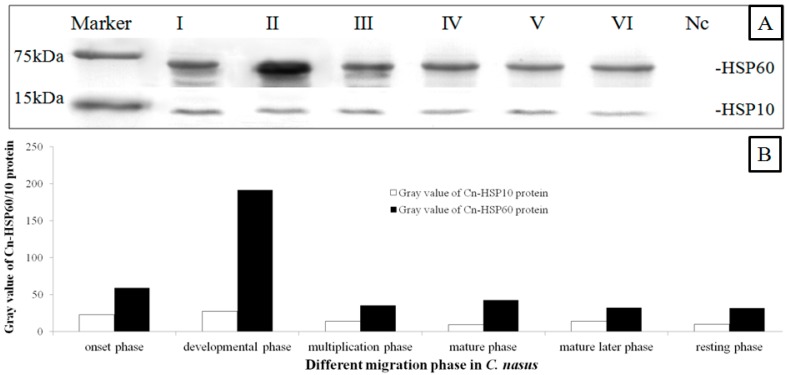
HSP60 and HSP10 protein expression patterns in different migration phases. Three fish in the different developmental phases were used for western blot (WB). The crude protein extract of adult *Coilia nasus* testes were pooled and then the WB was done as described. Marker; I: onset phase; II: developmental phase; III: multiplication phase; IV: mature phase; V: mature later phase; VI: resting phase; Nc: Negative Control. (**A**): HSP60 and HSP10 protein expression patterns in different migration phase; (**B**): The results were semi-quantitated analyzed by ImageJ2x 2.1 program. The expression level of HSP60 was considerately higher than the level of HSP10. HSP60 level reached the peak in the developmental phase. With the testes maturing, both proteins presented declining.

Anti-HSP60, anti-HSP10 and anti-serum are also recognized components in the crude protein extract of adult *C. nasus* testes. When the crude protein extracts were transferred to a nitrocellulose membrane and immunoblotted with anti-HSP60 and anti-HSP10, similar bands were observed on the immunoblot in the different phases of the migration process (Figure 4A). Control serum from the pre-immunized rabbit did not reveal recognition of any protein component in *C. nasus* testes extracts. The expression level of HSP60 was considerately higher than the level of HSP10. HSP60 levels were initially increasing and then declining, while HSP10 appeared declining continuously. Compared with other phases, HSP60 level reached the peak in the developmental phase. With the testes maturing, both proteins started to decline (Figure 4B).

### 2.5. Localization of HSP60/HSP10

**Figure 5:**
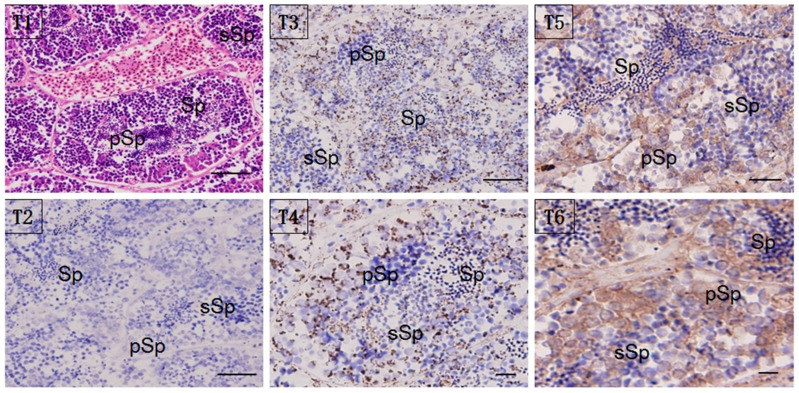
Localization of HSP60/HSP10 in the mature testis. Immunohistochemical (IHC) positive signals of HSP60/HSP10 immunolabeling are shown in brown. (**T1**): the whole testis section stained with H&E; (**T2**): negative control (NC); (**T3**): different part and developmental phase of testis for IHC with anti-HSP60; (**T4**): magnify for the IHC with anti-HSP60; (**T5**): different part and developmental phase of testis for IHC with anti-HSP10; (**T6**): magnify for the IHC with anti-HSP10, respectively. pSp: primary spermatocytes, sSp: secondary spermatocyte, and Sp: spermatids. Scale bar = 100 um.

Localization of the HSP60/HSP10 protein was studied by immunohistochemistry (IHC) and immunoelectron microscopy (IM). Whole sections of testis stained with hematoxylin-eosin (H & E) and with anti-HSP60/HSP10 immunolabeling (counterstained with H & E) were shown in Figure 5. Immunoreactive positive signals (in brown) for the HSP60/HSP10 protein were detected in the normal mature testis. Furthermore, the strongest signal for HSP60/HSP10 protein was also found in the primary spermatogonia, and with the lower signal in the secondary spermatocyte and mature sperm. Moreover, the HSP10 protein was more widely distributed in the testis sperm cells than the HSP60’s distribution. Interestingly, the HSP60 and HSP10 protein mainly distributed both in the sperm cytoplasm of different types, especially obvious in the developmental spermatocyte. There are no positive signals in the negative control, which was incubated with pre-immune rabbit serum (Figure 5(T2)).

**Figure 6:**
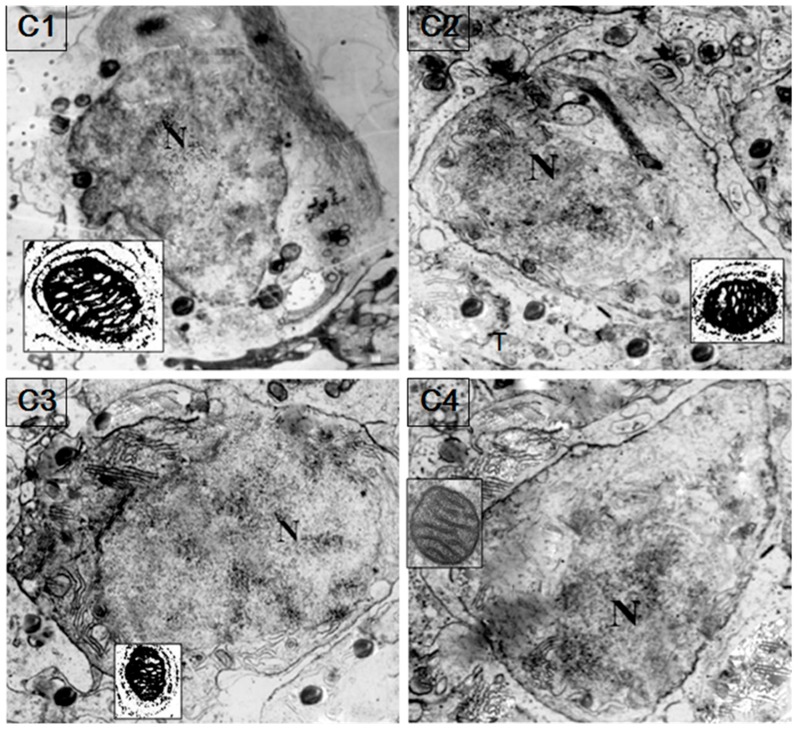
Colloidal gold immunocytochemical detection of HSP60/HSP10 in the sperm mitochondria. Colloidal gold particles are mainly present in the sperm mitochondrial inner membranes. The black dots show the immunolabeled mitochondria matrix. The little box shows the magnified figure of the immunolabeled mitochondria. N: nucleus. (**C1**): secondary spermatocyte incubated with anti-HSP60 (×5600); (**C2**): secondary spermatocyte incubated with anti-HSP60 (×5600); (**C3**): secondary spermatocyte incubated with anti-HSP10 and anti-HSP60 (×5600); (**C4**): secondary spermatocyte for the negative control with omission of the first antibody (×6600).

HSP60/HSP10 protein was found obviously in the sperm mitochondria and probably mainly on the mitochondrial inner membrane of the spermatozoa (Figure 6). Immunogold labeling of fish testis showed a specific accumulation of gold particles in the germ cell mitochondria matrix. Furthermore, HSP60/HSP10 immunoreactivity was not observed on other parts of spermatozoa or in negative preparations with the omission of the first antibody.

## 3. Discussion

In the present study, the complete cDNA sequence and characteristics of *HSP60* and *HSP10* genes in *C. nasus* were reported. These HSPs were found to be similar to their homologues from other fish species, and their deduced proteins had conserved characteristic family domains or motifs, which are in accordance with structures described in other species [10,22,23,24]. In the C-terminal tail, *Cn-HSP60* had flexible GGM repeats, which has been thought to play a specific role in facilitating the rearrangement of certain folding intermediated by providing a mildly hydrophobic, interactive surface [10,25]. Additionally, due to the fact that a highly conserved function is performed by a small polypeptide, the previously described lack of characteristic features of any *HSP10* was also found in the *C. nasus* sequence [23]. The mitochondrial targeting sequences were found in *Cn-HSP60/HSP10* (Figure 1A,B), which is similar to *Trypanosoma cruzi* HSP10 and other mitochondrial HSPs [26], and is involved in ATP hydrolysis, ATPase activity, and interdomain interaction. Numerous phosphorylation sites were also detected, which suggested that both *HSP10* and *HSP60* genes are functional. The deduced amino acid sequences of HSP10 and HSP60 shared the highest identity with sequences of *Clupea harengus* and *Oryzias latipes*, which suggested that these structure domains may be essential components in developmental biological and other physiological processes, such as spawning migration [27].

As expression pattern analysis showed (Figure 3A,B), *HSP60* and *HSP10* mRNA transcripts were ubiquitously expressed in all tissues examined and with higher expression in gonads. These results indicated that *HSP10* and *HSP60* were synthesized constitutively to support the basic metabolism and gonads development [8,28]. Moreover, the expression of *HSP10* and *HSP60* mRNA transcripts was significantly up-regulated during the onset of migration behavior (Figure 3C). When *C. nasus* spawning migration started, the water temperature (Tm) is in the wide range variation (Tm = 13.8–18.6 °C) and increasing by the migration process, so that both *HSP10* and *HSP60* mRNA transcripts were constitutively up-regulated to support the basic metabolism and spawning of *C. nasus*. These findings supported that both *HSP10* and *HSP60* are constitutive and inducible in the migration process and closely related with Tm change. *HSP10* and *HSP60* mRNA transcripts were up-regulated sharply and the highest level was observed in the development phase, which may imply that *HSP60* and *HSP10* may be essential promoter genes in mediating anadromous fish migration behavior. During *C. nasus* spawning migration, fish will encounter lots of stresses, such as thermal change and rising tide; it was seen that not only does the constitutive form of HSP60 and HSP10 accumulate (Figure 4A,B), but also the HSP60 and HSP10 matrix proteins are also upregulated (Figure 5 and Figure 6). These expression analysis results indicated that HSP60 shows a cell-type-specific and developmentally regulated expression pattern in the fish testis. In rat spermatogenesis, mitochondrial HSP60 is specifically expressed in spermatogonia and early spermatocyte, but not in the postmeiotic spermatids or spermatozoa [14,29]. Similar types of differential gene expression during rat spermatogenesis have been described for cytoplasmic HSP70 [30], and HSP90 [31]. In the gonadal somatic cells, expression of HSP60 was functionally specific for Leyding cells of the testis, and theca and corpora lutea cells of the ovary [14].

A previous study showed that HSP60 and HSP10 are essential for various cellular processes [28]. In this study, IHC results revealed that there were higher expression levels of HSP60 and HSP10 in the primary spermatogonia, weak or absent expression was seen in the mature germ cells (Figure 6). These results showed that the protein exists in abundance in germ cells from the beginning of the meiotic phase, which indicated that it is essential for germ proliferation and differentiation [18,28,30]. These findings are also in agreement with data from mammal species and suggest that the HSP60 and HSP10 proteins are primarily needed during the initial steps of gametogenesis and migration behavior [18,28]. Moreover, the presence of high levels of HSP60 and HSP10 in these phases (i.e., germ cell developmental and proliferative phase) may be caused by a very active cytoplasmic protein assembling machinery in which additional proteins such as HSP60/10 are needed for cell division [12,14]. Western blot (WB) results demonstrated that there are higher levels of HSP60/10 protein in the testes in the development phase (Figure 4). In the *C. nasus* spermatids, as the IM results showed, condensed mitochondria with a diffuse and vacuolated matrix are found (Figure 6), which is similar to the matrix-rich mitochondria of rat fetal and postnatal spermatogonia [14]. This makes biological sense in the process of protein assembly and import into the mitochondria, and therefore these proliferating spermatogenic cells would require more HSP60 to accommodate the generation of new mitochondria for daughter germ cells [9,27,32]. During spermatogenesis, mitochondria of spermatogenic cells must undergo rapid morphological changes. Therefore, it is possible that HSP60 and HSP10 modulate spermatogenesis in *C. nasus* by increasing the mitochondria matrix number and its activity in germ cells; and also perhaps when the fish begin to migrate lots of matrixes are generated so that the HSP60 and HSP10 expression is upregulated sharply [12,14]. These results indicated that HSP60 and HSP10 are very inducible to the spermatogenesis onset but not necessary to the sperm maturation.

How the germ cells adapt to their new environment and initi­ate and regulate migratory behaviors, are perplexing questions [11]. As for HSPs, it perhaps appears that they are at least thermally inducible and ubiquitous [24]. In *C. nasus*, the migration process begins with the water temperature rising; at this point the expression of HSP10 and HSP60 was significantly upregulated both in transcriptional and protein levels (Figure 4, Figure 5 and Figure 6). However, there was no obvious increase among the later migration phases (after the developmental phase); this result also supported that HSP10, HSP60 and their matrixes are inducible and functional in the migration process or spermatogenesis. During fish migration or spermatogenesis, prespermatogonia become separated from the vascular interstitium by the basement membrane and a layer of polarized supporting cells; expression of cytoplasmic HSP10 precedes that of the mitochondrial HSP60 [32]. According to the present concept, HSP60 has to be expressed and imported into mitochondria to ensure correct folding and assembling of denatured proteins during their import [33,34]. It is likely that the accumulation of cytoplasmic HSP10 and mitochondrial HSP60 reflects highly synthetic activity and protein transport [27]. Arrangement of the matrix-rich mitochondria around the spermatogonia nucleus may further be indicative of an energy-requiring transport of molecules between the germ cell nucleus and cytoplasm [10]. Furthermore, a particular set of HSPs’ accumulation may have an important role in the protection of germ cells against protein denature under physiological stress during their passage into new developmental pathways [14,27].

During the fish spawning migration, factors which regulate the development of the germ cells are still poorly known [11]. Our results on the expression of HSP60 and HSP10 expand and complement earlier reports of germ-cell-specific gene expression [9]. Even though HSP60/HSP10 matrix activity has been recognized for years, its contribution to germ cell development is still unresolved [18]. HSP60 is also involved in nascent or stress-denatured protein folding, but this function is based on forming a dual-ringed tetradecamer and requiring a lid-like heptamer cochaperonin protein complex HSP10 [17]. In the pubertal and mature rat testis, HSP60 protein and mRNA cannot be detected in the postmeiotic spermatids and spermatozoa [14]. The present results confirm the reports that HSP60 and HSP10 protein is no longer detectable by IHC during the haploid phase of spermatogenesis (i.e., mature sperm), and may also be because HSP60 and HSP10 transported into sperm cell nucleus [14,15]. Reduction of HSP60 and HSP10 expression to undetectable levels is parallel to the overall decrease in gene transcription and translation level during spermatogenesis or fish migration [35,36]. Interestingly, during rodent spermatogenesis, either in the mitochondria or cytoplasm, several mitochondrial genes are apparently active in the rodent sperm midpiece [37]. Further, as we know that spermatogenesis is characterized by the cardinal inflammatory reaction, mitochondrial and nuclear HSPs could have a role in the maintenance of the metabolic activity and survival of the sperm [28,38].

In conclusion, we characterized *HSP10* and *HSP60* molecular structure and expression patterns and found that HSP10 and HSP60 were inducible and functional in the *C. nasus* testis development and migration process, suggesting their essential roles in this process. The results also provided an expanded perspective on the collective roles of HSP families. Furthermore, this finding may be of vital importance for the development of gene markers to facilitate the developmental spermatogenesis or migration mechanism in *C. nasus*.

## 4. Material and Methods 

### 4.1. Fish Sampling and Organ Collection

Using a drift net, live healthy fish were sampled. Six populations of *C. nasus* were collected from six reaches in the Yangtze River during the anadromous period (from March to June, 2016), as described by Zhou and Fang [9,39]. All fish were field dissected to identify the developmental period and then we collected fishes which we needed. The approximate sampling time was determined through our annual fishery resources survey. *C. nasus* were caught by fisherman. *C. nasus* in the same development period in one of the sampling sites were sampled and different tissues (including the blood, brain, gill, liver, stomach, intestine, testis and ovary) were removed surgically (finished in 30 s) and then transferred to the laboratory in dry ice boxes, after that all tissues were stored at −80 °C until used. Testes were classified to six different phases according to the gonad development [40,41]. In short, six different phases were defined as follows: onset phase (Chongming section in March, Tm = 13.8–18.6 °C), developmental phase (Nantong section in March to April, Tm = 16.6–23.5 °C), multiplication phase (Jingjiang section in April to May, Tm = 21.3–24.2 °C), mature phase (Zhenjiang section in May, Tm = 21.6–23.7 °C), mature later phase (Dangtu section in late May to early June, Tm = 22.7–25.2 °C), and resting phase (Anqing section in mid to late June, Tm = 23.5–27.2 °C). Fish in each reproductive phase were collected in the field and stored at −80 °C for total RNA extraction and further experiments. All fish experimental procedures were approved and authorized by the Yangtze River Fish Committee in China. All experiments were performed in accordance with relevant guidelines and regulations.

### 4.2. Nucleic Acid Preparation

Total RNA was extracted from different tissues using an RNA Extraction kit reagent (Invitrogen, Carlsbad, CA, USA) according to the manufacturer’s protocol. The extracted RNA quality and concentration were identified by agarose gel electrophoresis and spectrophotometry, respectively. Total RNA (about 2 μg) was reverse transcribed using the SMART™ cDNA kit (Clontech, Mountain View, CA, USA) for cDNA cloning, and using the Prime Script^TM^ RT-PCR Kit (TaKaRa, Dalian, China) for semi-quantitative reverse transcriptase RT-PCR (RT-PCR) analysis or the PrimeScript Real-time PCR Kit (TaKaRa, Dalian, China) for real-time quantitative RT-PCR (qPCR) analysis respectively. Target fragments of *HSP60* and *HSP10* were obtained from our constructed transcriptome library after using BLAST programs at the National Center for Biotechnology Information [42]. Based on the target and cloning sequence, all the primers were designed by Primer Premier 5.0 and synthesized by Shanghai Bosun Biotech Co Ltd. (Bosun, Shanghai, China) (Table 1).

### 4.3. Gene Cloning of HSP60 and HSP10

The full-length sequences of *HSP60* and *HSP10* cDNA were obtained through RACE technology. The RACE reactions were performed using the SMARTer™ RACE cDNA amplification kit (Clontech, Mountain View, CA, USA) according to the kit protocol. Two pairs of gene-specific primers for *HSP60* and *HSP10* (GpHSP60-5’, GpHSP60-3’; GpHSP10-5’, GpHSP10-3’; Table 1) were used to obtain the full-length cDNA sequences. The PCR program was performed according to the RACE amplification kit protocol. The amplified cDNA fragments were cloned into the PMD18-T vector (TaKaRa, Dalian, China), and recombinants were identified by blue/white screening and confirmed by RT-PCR. Plasmids containing the inserted *HSP60/HSP10* fragment were used as the template for DNA sequencing. The sequences obtained were verified and analyzed comparing with HSP60 and HSP10 sequences in our unpublished genome data.

### 4.4. Analysis for Expression Patterns

Organ-dependent expressions of *HSP60* and *HSP10* mRNA were measured by RT-PCR and qPCR by the following method. Briefly, first-strand cDNA was prepared as described above. Gene-specific primers (HSP60-F, HSP60-R; HSP10-F, HSP10-R, Table 1) were designed based on the cloned *HSP60* and *HSP10* cDNA to produce an amplicon of 384 bp and 222 bp, respectively. All PCR reactions were performed in triplicate using extracted RNA of the same concentration (pooled RNA, *n* = 3, one in each migration phase, total *n* = 6 × 3 = 18 for RT-PCR; pooled RNA, *n* = 3 in each migration phase, total *n* = 3 × 6 = 18 for qPCR). Samples were also run in triplicate and normalized to the selected control gene *18sRNA*. The primers 18sRNA-R and 18sRNA-F were designed based on the *C. nasus* 18sRNA to amplify a fragment 232 bp. RT-qPCR was performed in a C1000™ Thermal Cycler (BioRad CFX 96™ Real-Time System) according to the manufacturer’s instructions. The final volume of each RT-qPCR reaction was 40 μL, which contained 20 μL SYBR Premix ExTaq (TaKaRa, Dalian, China), 2 μL of diluted cDNA as template, 17 μL of PCR-grade water, and 1μL of each 10 μM primer. PCR conditions were as follows: 98 °C for 30 s, followed by 40 cycles of 95 °C for 5 s and 58 °C 30 s. Gene mRNA transcripts expression levels were calculated by the 2^−ΔΔCT^ comparative CT method. Data were analyzed using the CFX Manager™ software version 1.6 (Bio-Rad, Foster City, CA, USA).

### 4.5. Western Blotting

The production of a synthetic peptide and monoclonal antibody was carried out commercially by Hua’an (Hua’an Biotech Co Ltd., Hangzhou, China). Briefly, a synthetic C-terminal peptide (TEIPKEEKEGGM for anti- HSP60; YFLFRDADILGKYVD for anti- HSP10) conjugated with keyhole limpet hemocyanin was emulsified with complete (for first immunization) and incomplete (for second to fourth) Freund adjuvant, and then injected into a New Zealand rabbit at intervals of 2–3 weeks. Before immunization and after the third and fourth injections, the rabbit was bled, and serum samples were collected. An increase in antibody titers against the peptide was verified by enzyme-linked immunosorbent assay (ELISA). Polyacrylamide gel electrophoresis (PAGE) of polypeptides was carried out in 12.5% gels in the presence of sodium dodecyl sulphate (SDS). The crude protein extracts of adult *C. nasus* testes in different developmental phases were pooled and then the WB was performed as described (*n* = 3, total *n* = 3 × 6 = 18). Testes were separated under a dissecting microscope in cold phosphate-buffered saline (PBS) containing 25 mM EDTA and 1 mM phenylmethyl sulfonylfluoride (PMSF) (Roche, Shanghai, China), respectively. Testes were washed in cold 10 mM Tris-HCl (4 °C, pH 7.4), dissolved in electrophoresis sample buffer and run through the polyacrylamide gels [43]. Gel portions were transferred to nitrocellulose membrane as described [14]. For immunoblotting, a blocking solution of 0.3% bovine serum albumin (BSA) in PBS was used. Incubation with the primary antibodies diluted 1:800 in PBS-BSA was followed by treatment with swine anti-rabbit Ig (1:100) and detected with the diaminobenzidine (DAB) method. Imbedded membranes were detected and gray values were analyzed by the software Image J2x 2.1. The average gray value was used to analyze the protein difference expression.

### 4.6. Immunohistochemistry (IHC)

Three male individuals were sampled and stored as described above for IHC analysis in the mature phase (*n* = 3). Frozen sections were used for analysis. Testes were removed from the fish by dissection and fixed in 0.01 M PBS containing 4% paraformaldehyde at 4 °C for 6 h. After washing with PBS three times, the samples were dehydrated in 30% saccharose-PBS solutions for 4 h at room temperature, and then embedded in organ optimal cutting temperature compound (Sakure, Los Angeles, CA, USA). Standard frozen sections of 8 μm in thickness were taken using a microtome (Leica, Bensheim, Germany). IHC was carried out as follows: briefly, after washing with 0.01 M PBS three times for 10 min each wash, sections were immersed in 0.01 M citric acid buffer (pH 6.0) containing 0.1% Tween 20, and autoclaved for 8 min. Then sections were treated in a blocking solution (Roche, Shanghai, China), incubated with anti-HSP60 (1:300) and anti-HSP10 (1:200) overnight at 4°C, and rinsed with 0.01 M PBS three times for 5 min each wash. Subsequently, the organ sections were incubated with goat anti-rabbit IgG conjugated with horseradish peroxidase for 30 min, and then rinsed with PBS three times for 5 min each wash. Immunoreactive signals were visualized using DAB (Sigma, Shanghai, China) as the substrate. Sections were counterstained with H & E. Organ sections were also incubated with pre-immune rabbit serum and the blocking solution as the negative control.

### 4.7. Immunoelectron Microscopy (IM)

For ultrastructural localization of anti-HSP60 and HSP10 immunoreactive epitomes, freshly isolated testes in the mature phase were used (*n* = 3). Tissues were fixed for 4 h in the fixative containing 0.05% glutaraldehyde and 4% paraformaldehyde in 0.05 M phosphate buffer (pH 7.2). After dehydration in 70% ethanol, tissues were incubated in 2:1, 1:1 and then 1:2 mixture (*v/v*) of ethanol and LR-White resin (Absin, Shanghai, China), each for 30 min at room temperature. Samples were immersed three times for 1 h in pure LR-White resin and thereafter, polymerization was carried out at 4°C for 5 days under UV illumination. Ultrathin sections were collected on form-vacated nickel grids and incubated with 0.5% egg albumin, 2% milk powder in PBS for 20 min. After incubation in the presence of HSP60 and HSP10 antibodies (1:10) for 1 h at room temperature, sections were washed in 1% BSA (Head, Beijing, China), 0.05% Triton X-100 and 0.05% Tween-20 in PBS (pH 7.4) and then incubated with anti-IgG-conjugated gold particles (5 and 20 nm, 1:40, Head, Beijing, China) for 1 h. The sections were counterstained with uranylacetate for10 min and examined under a Zeiss EM902 electron microscope.

### 4.8. Statistical Analysis

Data is given as mean ± one standard error (SE). Statistical significance was determined by one-way ANOVA, and post-hoc Duncan multiple range tests. Significance was set at *p* < 0.05.

## Figures and Tables

**Table 1:** Sequences of primers used in the present study.

Primer Name F—Forward/R—Reverse	DNA-Sequence 5′-3′	Annealing Temperature (°C)	Fragment Size (bp)
Gene-specific Primer pairs for RACE (GP)			
GpHSP60-5′	5′-CTCCACCCCTGCGTTCTTGGCTATCG-3′	74.4	-
GpHSP60-3′	5′-ATGACGATAGCCAAGAACGCAGGGGT-3′	65.5	-
GpHSP10-5′	5′-CCATTTCTTACGTTAAGAAATCAGG-3′	69.3	-
GpHSP10-3′	5′-TTTCATCCGCATCCAAGCAGACAAGA-3′	70.8	-
Primers for RT-qPCR			
HSP60-F	5′-GAATAACACCAACGAAGAGGCGG-3′	73.3	384
HSP60-R	5′-TTGATGAAGTATGGGGAGATGTAGCC-3′	64.9	
HSP10-F	5′-GTGGCAGTGGGTCCTGGTTCCGTC-3′	62.7	222
HSP10-R	5′-TCTTGTCTGCTTGGATGCGGATGA-3′	70.8	
18sRNA primers			
18sRNA-R	5′-TGATTGGGACTGGGGATTGAA-3′	59.2	232
18sRNA-F	5′-TAGCGACGGGCGGTGTGT-3′	62.4	
